# Minor alleles of *FTO* rs9939609 and rs17817449 polymorphisms confer a higher risk of type 2 diabetes mellitus and dyslipidemia, but not coronary artery disease in a Chinese Han population

**DOI:** 10.3389/fendo.2023.1249070

**Published:** 2023-12-15

**Authors:** Youjin Zhang, Lvlin Chen, Junchen Zhu, Hao Liu, Luying Xu, Yang Wu, Chuan He, Yongyan Song

**Affiliations:** ^1^ Central Laboratory, Clinical Medical College & Affiliated Hospital of Chengdu University, Chengdu, Sichuan, China; ^2^ Department of Critical Care Medicine, Clinical Medical College & Affiliated Hospital of Chengdu University, Chengdu, Sichuan, China; ^3^ Clinical Medical College of Chengdu University, Chengdu, Sichuan, China; ^4^ Department of Cardiology, Clinical Medical College & Affiliated Hospital of Chengdu University, Chengdu, Sichuan, China

**Keywords:** *FTO*, *PPARD*, polymorphism, T2DM, dyslipidemia

## Abstract

**Background:**

Relationships of the polymorphisms in fat mass and obesity-associated gene (*FTO*) and peroxisome proliferator-activated receptor delta gene (*PPARD*) with metabolic-related diseases remain to be clarified.

**Methods:**

One thousand three hundred and eighty-one subjects were enrolled. Metabolic-related diseases including obesity, dyslipidemia, hyperhomocysteinemia, hyperuricemia, hypertension, type 2 diabetes mellitus (T2DM) and coronary artery disease (CAD) were defined based on diagnostic criteria. *FTO* rs9939609 and rs17817449, and *PPARD* rs2016520 and rs2267668 polymorphisms were genotyped by using polymerase chain reaction-restricted fragment length polymorphism method.

**Results:**

Patients with T2DM or dyslipidemia had a higher frequency of AA, AT or AA + AT genotypes as well as A allele of *FTO* rs9939609 polymorphism than those free of T2DM or dyslipidemia (*P* ≤ 0.04 for all). Patients with T2DM or dyslipidemia had a higher frequency of GG, GT or GG + GT genotypes as well as G allele of *FTO* rs17817449 polymorphism than those free of T2DM or dyslipidemia (*P* ≤ 0.03 for all). Multivariate logistic regression analyses showed that *FTO* rs9939609 and rs17817449 polymorphisms were independently associated with T2DM as well as dyslipidemia after adjustment for age, sex, smoking and other metabolic diseases. *FTO* rs9939609 and rs17817449 polymorphisms were not associated with obesity, hyperhomocysteinemia, hyperuricemia, hypertension and CAD. Obese or T2DM carriers of the AA or AT genotype of the *FTO* rs9939609 polymorphism had a higher prevalence of dyslipidemia compared to non-obese or non-T2DM carriers of the AA or AT genotype (*P* = 0.03 for both). Among the carriers of GG or GT genotype of the *FTO* rs17817449 polymorphism, the prevalence of dyslipidemia in obese patients was higher than that in non-obese subjects (*P* < 0.01). *PPARD* rs2016520 and rs2267668 polymorphisms were not correlated with any of the metabolic-related diseases in the study population.

**Conclusion:**

Minor alleles of *FTO* rs9939609 and rs17817449 polymorphisms confer a higher risk of T2DM and dyslipidemia, and the risk is further increased among obese individuals. *PPARD* rs2016520 and rs2267668 polymorphisms are not associated with metabolic-related diseases.

## Introduction

Metabolic-related diseases and chronic conditions, such as obesity, diabetes, dyslipidemia, hyperuricemia and hyperhomocysteinemia, can occur in people of all ages and confer a very high risk of developing life-threatening cardiovascular and cerebrovascular diseases (1). Metabolic-related diseases are a group of multifactorial diseases with a number of risk factors including genetic susceptibility, sedentary lifestyle, high-fat diet, old age, cigarette smoking, and alcohol drinking. Genetic susceptibility may be the most important risk factor for metabolic-related diseases (2, 3). Researchers around the world have paid a great effort to investigate the relations of genetic variants with metabolic-related diseases in the past few decades, but the findings were inconsistent and inconclusive due to various reasons such as small sample size and differences in race, dietary habit, health condition, and social and natural environments across the study populations (4–7).

Fat mass and obesity-associated gene (*FTO*) encodes a N6-methyladenosine (m6A) demethylase which belongs to the superfamily of 2-oxoglutarate- and Fe(II)-dependent dioxygenases. The main function of FTO is to remove m6A from specific RNA species, such as mRNAs, thereby affecting the stability of RNAs, translation efficiency of mRNAs or binding activity of m6A readers (8, 9). FTO mostly targets the mRNAs that are closely related to glucolipid metabolism and energy homoeostasis, such as sterol regulatory element binding protein 1c (SREBP1c) (10–12), carbohydrate responsive element binding protein (ChREBP) (12), peroxisome proliferator-activated receptor alpha (PPARα) (13), PPARγ (14), PPARγ coactivator 1α (PGC1α) (15), apolipoprotein E (APOE) (16), uncoupling protein 1 (UCP1) (17) and ghrelin (18). Based on the functions of the target mRNAs of FTO, it is logical to speculate that the genetic variations in *FTO* are involved in the occurrence and development of metabolic-related diseases. Human *FTO* is located on chromosome 16q12.2 and consists of 9 exons and 8 introns. The rs9939609 (g.53786615T>A) and rs17817449 (g.53779455T>G) variants are localized within intron 1 of *FTO*, and rs17817449 is positioned approximately 7.2 kb upstream of the rs9939609 polymorphism. The rs9939609 polymorphism is formed by a transversion from thymine (T) to adenine (A), while the rs17817449 polymorphism is formed from thymine (T) to guanine (G). These two loci have been reported to be associated to metabolic-related diseases such as obesity (19–23), dyslipidemia (22, 24), hypertension (22, 25–27), type 2 diabetes mellitus (T2DM) (28, 29) and cardiovascular disease (20, 27, 30). However, some other investigators have come to completely different and even conflicting conclusions, demonstrating that the minor alleles of these genetic variants were either not associated or negatively associated with metabolic-related diseases (31–33).

PPARδ (also known as PPARβ) is a transcription factor and an important member of the PPAR family of nuclear receptors, with the other two members PPARα and PPARγ. As a nuclear transcription factor, PPARδ mainly initiates transcription of the genes related to lipid metabolism (34–36), adaptive thermogenesis (37) and adipocyte formation (38). Human PPARδ gene (*PPARD*) is located on chromosome 6p21.3 and contains 8 exons and 7 introns. The rs2016520 and rs2267668 polymorphisms are positioned in the 5’ untranslated region (5’UTR) and intron 2 of *PPARD*, respectively. The rs2016520 variant is also known as +294T/C, -87T/C or +15T/C, with cytosine (C) as its minor allele. The rs2267668 polymorphism is localized 856 bp upstream of the rs2016520 variant. The two polymorphic loci have been proved to be related to metabolic-related diseases such as obesity (39) and dyslipidemia (40). However, several other studies did not support these findings (41, 42).

The relationships of *FTO* and *PPARD* variants with metabolic-related diseases deserve to be further explored since there were so many inconsistencies in the scientific publications. Moreover, the associations of the four polymorphisms with hyperuricemia and hyperhomocysteinemia have not yet been investigated to date. Here, a hospital-based study with the clinical diagnosis of metabolic-related diseases or chronic conditions including obesity, dyslipidemia, hypertension, hyperhomocysteinemia, hyperuricemia, T2DM and coronary artery disease (CAD) was conducted to systematically explore the relations of *FTO* rs9939609 and rs17817449, and *PPARD* rs2016520 and rs2267668 polymorphisms with metabolic-related diseases. The results from this study can provide an opportunity to unveil the interrelationships among variants in *FTO* and *PPARD*, metabolic disorders and CAD.

## Subjects and methods

### Subjects

A total of 1381 unrelated Chinese adult subjects who underwent coronary angiography and other examinations for suspected CAD at the Department of Cardiology, Affiliated Hospital of Chengdu University (Chengdu, China) were enrolled in the study between July 2021 and December 2022. The exclusion criteria were as follows: 1) subjects with renal, hepatic or valvular disease; 2) subjects who were taking antihypertensive drugs; 3) subjects taking the drugs that may influence plasma glucose, lipid, uric acid or homocysteine level. Subjects who were taking the drugs that were considered not to affect plasma glucose, lipid, uric acid or homocysteine level were still included in this study to increase sample size and statistical power. Written informed consent was provided by all participants or their legal guardians prior to participation in the study. The study protocol was reviewed and approved by the Ethics Committee of Chengdu University. The study adhered to the tenets of the Declaration of Helsinki.

### Diagnosis of metabolic-related diseases

Smoking was defined as current smoking or having ever smoked cigarettes. T2DM was defined as fasting serum glucose level exceeding 7.0 mmol/L (126 mg/dL), or regular use of anti-diabetic medications. Dyslipidemia was defined as the presence of one or more of the following conditions: triglyceride (TG) ≥ 2.3 mmol/L, total cholesterol (TC) ≥ 6.2 mmol/L, low-density lipoprotein cholesterol (LDL-C) ≥ 4.1 mmol/L and high-density lipoprotein cholesterol (HDL-C) < 1.0 mmol/L (43). Serum glucose, TG, TC, LDL-C and HDL-C were measured by using enzymatic method, and all measurements were performed with an automatic clinical chemistry analyzer (Beckman Coulter AU5800, USA). CAD was diagnosed if one or more major coronary arteries (i.e., left main coronary artery, left anterior descending artery, left circumflex artery and right coronary arteries) had stenosis greater than 50%. Coronary angiography was conducted by using Allura Xper FD20 (Philips Medical Systems Nederland B.V. Netherlands) with at least two views of right coronary arteries and four views of the left coronary system. Obesity was defined as body mass index above 28 kg/m^2^ (44). Hypertension was diagnosed if systolic/diastolic blood pressure is greater than 140/90 mmHg or antihypertensive medications are regularly used (45). Hyperhomocysteinemia was defined as the fasting serum level of homocysteine exceeding 15 μmol/L. Hyperuricemia was defined as the fasting serum level of uric acid exceeding 7.3 mg/dL.

### Genomic DNA extraction and genotyping

Genomic DNA was extracted from peripheral lymphocytes by using a whole blood DNA isolation kit according to the instruction manual (QIAGEN, Beijing, China). The genotyping of *FTO* rs9939609 and rs17817449, and *PPARD* rs2016520 and rs2267668 polymorphisms was performed by using polymerase chain reaction-restricted fragment length polymorphism method. PCR was carried out in a 25 μL reaction system consisting of 0.03 ug genomic DNA, 12.5 uL 2 × Taq PCR Mix (Tiangen, Beijing, China, cat # KT211), 0.01 uM forward primer, and 0.01 uM reverse primer. Next, PCR products were digested by restriction endonucleases and genotypes were determined by the size of the digested DNA fragments. Three uL PCR products were digested with 5 U restriction endonucleases (NEB, Beijing, China), and the DNA fragments were separated on a 2%-3% agarose gel containing nucleic acid dye (Goldview) and visualized under UV light. The PCR primer sequences, product size, amplification conditions, restriction endonucleases and the DNA fragment sizes of the *FTO* and *PPARD* polymorphisms are shown in **Table S1**. The gels displaying the different fragments of the *FTO* and *PPARD* polymorphisms are presented in **Figures S1–S4**.

### Statistical analysis

Continuous data are expressed as mean and standard deviation. Differences between disease and disease-free groups, or among the subjects with different genotypes were analyzed by analysis of variance for continuous variables, and Chi-square test for categorical variables. Multivariate logistic regression analysis was carried out to analyze the associations between the *FTO* rs9939609 and rs17817449 polymorphisms and T2DM as well as dyslipidemia adjusting for possible confounding factors. The results of logistic regression analysis were presented as odds ratio (OR) and corresponding 95% confidence interval (CI). All *P* values were two-tailed and the differences were considered as significant if *P* ≤ 0.05. Statistical analysis was done by using 15.0 version SPSS (Chicago, IL, USA).

## Results

### Characteristics of the study population

Characteristics of the study population are shown in **Table 1**. The study population consisted of 858 men and 523 women, and women had an older average age than men (*P* < 0.001). The prevalence of smoking (*P* < 0.001), dyslipidemia (*P* < 0.001), hyperhomocysteinemia (*P* < 0.001), T2DM (*P* = 0.02) and CAD (*P* < 0.001) in men were higher than in women. The prevalence of obesity, hypertension and hyperuricemia was comparable between men and women.

**Table 1:** Characteristics of the study population.

Variables	Male (n = 858)	Female (n = 523)	*P*
Age, years	62.55 ± 11.99	65.05 ± 10.20	< 0.001
Smoking, n (%)	542 (63.17%)	11 (2.10%)	< 0.001
Obesity, n (%)	107 (12.47%)	66 (12.62%)	0.94
Dyslipidemia, n (%)	402 (46.85%)	193 (36.90%)	< 0.001
Hyperhomocysteinemia, n (%)	360 (41.96%)	113 (21.61%)	< 0.001
Hyperuricemia, n (%)	258 (30.07%)	139 (26.58%)	0.16
Hypertension, n (%)	484 (56.41%)	292 (55.83%)	0.83
T2DM, n (%)	218 (25.41%)	105 (20.08%)	0.02
CAD, n (%)	558 (65.03%)	239 (45.70%)	< 0.001

T2DM, type 2 diabetes mellitus; CAD, coronary artery disease.

### Associations of *FTO* rs9939609 and rs17817449 polymorphisms with metabolic-related diseases

As shown in **Table 2**, there was a rising trend in the frequencies of AA genotype, AT genotype, AA + AT genotypes and A allele of the rs9939609 polymorphism in patients with dyslipidemia (*P* ≤ 0.04 for all) and T2DM (*P* ≤ 0.001 for all). The prevalence of AA genotype, AT genotype, AA + AT genotypes and A allele of the rs9939609 polymorphism was 1.69%, 25.46%, 27.15% and 14.42% in dyslipidemia patients, as compared to 1.05%, 21.23%, 22.28% and 11.66% in dyslipidemia-free subjects, respectively (*P* ≤ 0.04 for all). The prevalence of AA genotype, AT genotype, AA + AT genotypes and A allele of the rs9939609 polymorphism was 2.79%, 28.48%, 31.27% and 17.03% in T2DM patients, as compared to 0.85%, 21.50%, 22.35% and 11.60% in T2DM-free subjects, respectively (*P* ≤ 0.001 for all). Associations of *FTO* rs17817449 polymorphism with metabolic-related diseases are shown in **Table 3**. The prevalence of GG genotype, GT genotype, GG + GT genotypes and G allele of the rs17817449 polymorphism was 2.02%, 27.32%, 29.34% and 15.68% in dyslipidemia patients, as compared to 1.32%, 22.80%, 24.12% and 12.71% in dyslipidemia-free subjects, respectively (*P* = 0.03 for all). The prevalence of GG genotype, GT genotype, GG + GT genotypes and G allele of the rs17817449 polymorphism was 3.10%, 29.41%, 32.51% and 17.80% in T2DM patients, as compared to 1.14%, 23.48%, 24.62% and 12.88% in T2DM-free subjects, respectively (*P* < 0.01 for all). However, *FTO* rs9939609 and rs17817449 polymorphisms were not associated with obesity, hyperhomocysteinemia, hyperuricemia, hypertension and CAD in this study population.

**Table 2:** Associations of *FTO* rs9939609 polymorphism with metabolic-related diseases.

Metabolic-related diseases	TT genotype	TA genotype	AA genotype	TA+AA genotypes	T allele	A allele
Obesity, n (%)	122 (71.35)	46 (26.90)	3 (1.75)	49 (28.65)	290 (84.80)	52 (15.20)
Obesity-free, n (%)	812 (76.24)	240 (22.54)	13 (1.22)	253 (23.76)	1864 (87.51)	266 (12.49)
*P*		0.16		0.17		0.16
Dyslipidemia, n (%)	432 (72.85)	151 (25.46)	10 (1.69)	161 (27.15)	1015 (85.58)	171 (14.42)
Dyslipidemia-free, n (%)	593 (77.72)	162 (21.23)	8 (1.05)	170 (22.28)	1348 (88.34)	178 (11.66)
*P*		0.03		0.04		0.03
Hyperhomocysteinemia, n (%)	362 (76.69)	107 (22.67)	3 (0.64)	110(23.31)	831 (88.03)	113 (11.97)
Hyperhomocysteinemia-free, n (%)	515 (73.68)	172 (24.61)	12 (1.71)	184(26.32)	1202 (85.98)	196 (14.02)
*P*		0.14		0.24		0.15
Hyperuricemia, n (%)	304 (76.96)	87 (22.03)	4 (1.01)	91 (23.04)	695 (87.97)	95 (12.03)
Hyperuricemia-free, n (%)	723 (75.08)	227 (23.57)	13 (1.35)	240 (24.92)	1673 (86.86)	253 (13.14)
*P*		0.42		0.46		0.43
Hypertension, n (%)	593 (76.52)	174 (22.45)	8 (1.03)	182 (23.48)	1360 (87.74)	190 (12.26)
Hypertension-free, n (%)	447 (74.50)	143 (23.83)	10 (1.67)	153 (25.50)	1037 (86.42)	163 (13.58)
*P*		0.30		0.39		0.30
T2DM, n (%)	222 (68.73)	92 (28.48)	9 (2.79)	101 (31.27)	536 (82.97)	110 (17.03)
T2DM-free, n (%)	820 (77.65)	227 (21.50)	9 (0.85)	236 (22.35)	1867 (88.40)	245 (11.60)
*P*		< 0.001		0.001		< 0.001
CAD, n (%)	596 (74.97)	186 (23.40)	13 (1.63)	199 (25.03)	1378 (86.67)	212 (13.33)
CAD-free, n (%)	446 (76.37)	133 (22.77)	5 (0.86)	138 (23.63)	1025 (87.76)	143 (12.24)
*P*		0.39		0.55		0.40

*FTO*, fat mass and obesity-associated gene; T2DM, type 2 diabetes mellitus; CAD, coronary artery disease.

**Table 3:** Associations of *FTO* rs17817449 polymorphism with metabolic-related diseases.

Metabolic-related diseases	TT genotype	TG genotype	GG genotype	TG+GG genotypes	T allele	G allele
Obesity, n (%)	117 (68.42)	50 (29.24)	4 (2.34)	54 (31.58)	284 (83.04)	58 (16.96)
Obesity-free, n (%)	791 (74.27)	258 (24.23)	16 (1.50)	274 (25.73)	1840 (86.38)	290 (13.62)
*P*		0.09		0.11		0.10
Dyslipidemia, n (%)	419 (70.66)	162 (27.32)	12 (2.02)	174 (29.34)	1000 (84.32)	186 (15.68)
Dyslipidemia-free, n (%)	579 (75.88)	174 (22.80)	10 (1.32)	184 (24.12)	1332 (87.29)	194 (12.71)
*P*		0.03		0.03		0.03
Hyperhomocysteinemia, n (%)	354 (75.00)	116 (24.58)	2 (0.42)	118 (25.00)	824 (87.29)	120 (12.71)
Hyperhomocysteinemia-free, n (%)	502 (71.82)	182 (26.04)	15 (2.14)	197 (28.18)	1186 (84.84)	212 (15.16)
*P*		0.09		0.23		0.10
Hyperuricemia, n (%)	295 (74.68)	96 (24.30)	4 (1.02)	100 (25.32)	686 (86.84)	104 (13.16)
Hyperuricemia-free, n (%)	704 (73.03)	242 (25.10)	18 (1.87)	260 (26.97)	1650 (85.58)	278 (14.42)
*P*		0.39		0.53		0.39
Hypertension, n (%)	575 (74.19)	191 (24.65)	9 (1.16)	200 (25.81)	1341 (86.52)	209 (13.48)
Hypertension-free, n (%)	437 (72.83)	150 (25.00)	13 (2.17)	163 (27.17)	1024 (85.33)	176 (14.67)
*P*		0.37		0.57		0.38
T2DM, n (%)	218 (67.49)	95 (29.41)	10 (3.10)	105 (32.51)	531 (82.20)	115 (17.80)
T2DM-free, n (%)	796 (75.38)	248 (23.48)	12 (1.14)	260 (24.62)	1840 (87.12)	272 (12.88)
*P*		0.001		< 0.01		< 0.01
CAD, n (%)	580 (72.96)	200 (25.16)	15 (1.88)	215 (27.04)	1360 (85.53)	230 (14.47)
CAD-free, n (%)	434 (74.32)	143 (24.49)	7 (1.19)	150 (25.68)	1011 (86.56)	157 (13.44)
*P*		0.44		0.57		0.44

*FTO*, fat mass and obesity-associated gene; T2DM, type 2 diabetes mellitus; CAD, coronary artery disease.

### Multivariate logistic regression analyses between the *FTO* rs9939609 and rs17817449 polymorphisms and T2DM as well as dyslipidemia

The results of multivariate logistic regression analyses between the *FTO* rs9939609 and rs17817449 polymorphisms and T2DM as well as dyslipidemia are shown in **Table 4**. The *FTO* rs9939609 polymorphism was independently associated with T2DM after controlling for the confounding variables including age, sex, smoking, obesity, dyslipidemia, hyperhomocysteinemia, hyperuricemia, hypertension and CAD (*P* < 0.01). The *FTO* rs9939609 polymorphism was independently associated with dyslipidemia after adusting for age, sex and smoking (*P* = 0.05), but the model lost its significance when the variables including obesity, dyslipidemia, hyperhomocysteinemia, hyperuricemia, hypertension and CAD were included (*P* = 0.12). The *FTO* rs17817449 polymorphism was independently associated with both T2DM (*P* = 0.04) and dyslipidemia (*P* = 0.05) after adjusting for the confounding variables including age, sex, smoking, obesity, dyslipidemia, hyperhomocysteinemia, hyperuricemia, hypertension and CAD.

**Table 4:** Multivariate logistic regression analysis between the *FTO* rs9939609 and rs17817449 polymorphisms and T2DM as well as dyslipidemia.

Models	Model 1		Model 2		Model 3	
	Adjusted OR (95% CI)	*P*	Adjusted OR (95% CI)	*P*	Adjusted OR (95% CI)	*P*
T2DM						
rs9939609	1.61 (1.25-2.08)	<0.001	1.61 (1.25-2.08)	<0.001	1.53 (1.14-2.05)	<0.01
rs17817449	1.51 (1.18-1.93)	0.001	1.50 (1.17-1.92)	0.001	1.37 (1.02-1.83)	0.04
Dyslipidemia						
rs9939609	1.25 (0.99-1.58)	0.06	1.26 (1.00-1.59)	0.05	1.24 (0.95-1.62)	0.12
rs17817449	1.26 (1.01-1.57)	0.045	1.27 (1.01-1.58)	0.04	1.29 (1.00-1.68)	0.05

Model 1, adjusted for age and sex; Model 2, adjusted for age, sex and smoking; Model 3, adjusted for age, sex, smoking, obesity, dyslipidemia (included when analyzing T2DM), hyperhomocysteinemia, hyperuricemia, hypertension, T2DM (included when analyzing dyslipidemia) and CAD. OR, odds ratio; 95% CI, 95% confidence interval; *FTO*, fat mass and obesity-associated gene; T2DM, type 2 diabetes mellitus; CAD, coronary artery disease.

### Associations of *PPARD* rs2016520 and rs2267668 polymorphisms with metabolic-related diseases

As shown in **Tables 5**, **6**, no significant associations of *PPARD* rs2016520 and rs2267668 polymorphisms with obesity, dyslipidemia, hyperhomocysteinemia, hyperuricemia, hypertension, T2DM and CAD were detected in the present study (*P* > 0.05 for all).

**Table 5:** Associations of *PPARD* rs2016520 polymorphism with metabolic-related diseases.

Metabolic-related diseases	TT genotype	TC genotype	CC genotype	TC+CC genotypes	T allele	C allele
Obesity, n (%)	93 (53.76)	68 (39.31)	12 (6.93)	80 (46.24)	254 (73.41)	92 (26.59)
Obesity-free, n (%)	558 (52.35)	422 (39.59)	86 (8.06)	508 (47.65)	1538 (72.14)	594(27.86)
*P*		0.63		0.73		0.62
Dyslipidemia, n (%)	320 (53.78)	233 (39.16)	42 (7.06)	275 (46.22)	873 (73.36)	317 (26.64)
Dyslipidemia-free, n (%)	392 (51.31)	308 (40.31)	64 (8.38)	372 (48.69)	1092 (71.47)	436 (28.53)
*P*		0.28		0.37		0.27
Hyperhomocysteinemia, n (%)	261 (55.06)	175 (36.92)	38 (8.02)	213 (44.94)	697 (73.52)	251 (26.48)
Hyperhomocysteinemia-free, n (%)	361 (51.57)	282 (40.29)	57 (8.14)	339 (48.43)	1004 (71.71)	396 (28.29)
*P*		0.34		0.24		0.34
Hyperuricemia, n (%)	219 (55.16)	152 (38.29)	26 (6.55)	178 (44.84)	590 (74.31)	204 (25.69)
Hyperuricemia-free, n (%)	494 (51.19)	389 (40.31)	82 (8.50)	471 (48.81)	1377 (71.35)	553 (28.65)
*P*		0.12		0.18		0.12
Hypertension, n (%)	408 (52.51)	306 (39.38)	63 (8.11)	369 (47.49)	1122 (72.20)	432 (27.80)
Hypertension-free, n (%)	314 (52.25)	242 (40.27)	45 (7.48)	267 (47.75)	870 (72.38)	332 (27.62)
*P*		0.92		0.58		0.92
T2DM, n (%)	165 (51.08)	128 (39.63)	30 (9.29)	158 (48.92)	458 (70.90)	188 (29.10)
T2DM-free, n (%)	558 (52.69)	423 (39.94)	78 (7.37)	501 (47.31)	1539 (72.66)	579 (27.34)
*P*		0.38		0.61		0.38
CAD, n (%)	421 (52.76)	316 (39.60)	61 (7.64)	377 (47.24)	1158 (72.56)	438 (27.44)
CAD-free, n (%)	302 (51.71)	235 (40.24)	47 (8.05)	282 (48.29)	839 (71.83)	329 (28.17)
*P*		0.68		0.70		0.67

*PPARD*, Peroxisome proliferator activated receptor delta gene; T2DM, type 2 diabetes mellitus; CAD, coronary artery disease.

**Table 6:** Associations of *PPARD* rs2267668 polymorphism with metabolic-related diseases.

Metabolic-related diseases	AA genotype	AG genotype	GG genotype	AG+GG genotypes	A allele	G allele
Obesity, n (%)	102 (59.30)	61 (35.47)	9 (5.23)	70 (40.70)	265 (77.03)	79 (22.97)
Obesity-free, n (%)	623 (58.50)	370 (34.74)	72 (6.76)	442 (41.5)	1616 (75.87)	514 (24.13)
*P*		0.65		0.84		0.64
Dyslipidemia, n (%)	345 (58.08)	212 (35.69)	37 (6.23)	249 (41.92)	902 (75.93)	286 (24.07)
Dyslipidemia-free, n (%)	442 (57.93)	268 (35.12)	53 (6.95)	321 (42.07)	1152 (75.49)	374 (24.51)
*P*		0.81		0.96		0.79
Hyperhomocysteinemia, n (%)	288 (60.89)	150 (31.71)	35 (7.40)	185 (39.11)	726 (76.74)	220 (23.26)
Hyperhomocysteinemia-free, n (%)	400 (57.22)	255 (36.48)	44 (6.30)	299 (42.78)	1055 (75.46)	343 (24.54)
*P*		0.49		0.21		0.48
Hyperuricemia, n (%)	231 (58.33)	144 (36.36)	21 (5.30)	165 (41.66)	606 (76.52)	186 (23.48)
Hyperuricemia-free, n (%)	556 (57.68)	338 (35.06)	70 (7.26)	408 (42.32)	1450 (75.21)	478 (24.79)
*P*		0.48		0.82		0.47
Hypertension, n (%)	452 (58.25)	268 (34.54)	56 (7.21)	324 (41.75)	1172 (75.52)	380 (24.48)
Hypertension-free, n (%)	343 (57.26)	221 (36.89)	35 (5.85)	256 (42.74)	907 (75.71)	291 (24.29)
*P*		0.91		0.71		0.91
T2DM, n (%)	186 (57.59)	112 (34.67)	25 (7.74)	137 (42.41)	484 (74.92)	162 (25.08)
T2DM-free, n (%)	611 (57.81)	380 (35.95)	66 (6.24)	446 (42.19)	1602 (75.78)	512 (24.22)
*P*		0.66		0.94		0.66
CAD, n (%)	464 (58.29)	277 (34.80)	55 (6.91)	332 (41.71)	1205 (75.69)	387 (24.31)
CAD-free, n (%)	333 (57.02)	215 (36.82)	36 (6.16)	251 (42.98)	881 (75.43)	287 (24.57)
*P*		0.88		0.64		0.87

*PPARD*, Peroxisome proliferator activated receptor delta gene; T2DM, type 2 diabetes mellitus; CAD, coronary artery disease.

### Interactions of *FTO* rs9939609 and rs17817449 polymorphisms with obesity and other factors on the risk of dyslipidemia and T2DM

There was a significant interaction between *FTO* rs9939609 polymorphism and obesity or T2DM on dyslipidemia risk (**Table 7**). The prevalence of dyslipidemia was higher in obese or T2DM carriers of AA or AT genotype of the rs9939609 polymorphism than in non-obese or non-T2DM carriers of AA or AT genotype (*P* = 0.03 for both). Interestingly, carriers of AA or AT genotype of the rs9939609 polymorphism had a marginally nonsignificantly lower incidence of dyslipidemia in smokers than those in nonsmokers (*P* = 0.07). There may also be an interaction between the rs9939609 polymorphism and obesity on T2DM risk, since obese carriers of AA or AT genotype of the rs9939609 polymorphism had a marginally insignificantly higher incidence of T2DM compared to non-obese carriers of AA or AT genotype (*P* = 0.09). Significant interaction between *FTO* rs17817449 polymorphism and obesity on dyslipidemia risk has been observed (**Table 8**). Obese carriers of GG or GT genotype of the rs17817449 polymorphism had a higher incidence of dyslipidemia than non-obese carriers of GG or GT genotype (*P* < 0.01). Similar to the rs9939609 polymorphism, carriers of GG or GT genotype of the rs17817449 polymorphism had a marginally insignificantly lower incidence of dyslipidemia in smokers than those in nonsmokers (*P* = 0.08). No other interactions on dyslipidemia or T2DM risk were detected in the present study.

**Table 7:** Interactions of *FTO* rs9939609 polymorphism with obesity and other factors on the risk of T2DM or dyslipidemia.

Factors	Dyslipidemia			T2DM		
	TT genotype	TA genotype	AA genotype	TT genotype	TA genotype	AA genotype
Male, n (%)	293 (73.07)	102 (25.44)	6 (1.49)	147 (67.43)	63 (28.90)	8 (3.67)
Female, n (%)	139 (72.40)	49 (25.52)	4 (2.08)	75 (71.43)	29 (27.62)	1 (0.95)
*P*		0.77			0.29	
Smoking, n (%)	196 (76.26)	59 (22.96)	2 (0.78)	96 (66.67)	43 (29.86)	5 (3.47)
Non-smoking, n (%)	234 (70.48)	90 (27.11)	8 (2.41)	126 (71.19)	47 (26.55)	4 (2.26)
*P*		0.07			0.34	
Obesity, n (%)	62 (64.58)	31 (32.29)	3 (3.13)	26 (57.78)	17 (37.78)	2 (4.44)
Obesity-free, n (%)	338 (75.28)	104 (23.16)	7 (1.56)	165 (69.92)	66 (27.97)	5 (2.11)
*P*		0.03			0.09	
Dyslipidemia, n (%)	–	–	–	109 (67.28)	48 (29.63)	5 (3.09)
Dyslipidemia-free, n (%)	–	–	–	112 (71.79)	40 (25.64)	4 (2.57)
*P*		–			0.40	
Hyperhomocysteinemia, n (%)	163 (73.42)	56 (25.23)	3 (1.35)	75 (70.09)	30 (28.04)	2 (1.87)
Hyperhomocysteinemia-free, n (%)	231 (71.30)	87 (26.85)	6 (1.85)	112 (67.47)	50 (30.12)	4 (2.41)
*P*		0.54			0.62	
Hyperuricemia, n (%)	151 (74.38)	49 (24.14)	3 (1.48)	66 (74.16)	20 (22.47)	3 (3.37)
Hyperuricemia-free, n (%)	276 (71.69)	102 (26.49)	7 (1.82)	154 (67.25)	69 (30.13)	6 (2.62)
*P*		0.48			0.35	
Hypertension, n (%)	247 (73.51)	83 (24.70)	6 (1.79)	152 (70.37)	58 (26.85)	6 (2.78)
Hypertension-free, n (%)	183 (71.76)	68 (26.67)	4 (1.57)	70 (65.42)	34 (31.78)	3 (2.80)
*P*		0.71			0.43	
T2DM, n (%)	109 (67.28)	48 (29.63)	5 (3.09)	–	–	–
T2DM-free, n (%)	323 (74.94)	103 (23.90)	5 (1.16)	–	–	–
*P*		0.03			–	
CAD, n (%)	279 (72.47)	98 (25.45)	8 (2.08)	154 (70.00)	58 (26.36)	8 (3.64)
CAD-free, n (%)	153 (73.56)	53 (25.48)	2 (0.96)	68 (66.02)	34 (33.01)	1 (0.97)
*P*		0.60			0.84	

*FTO*, fat mass and obesity-associated gene; T2DM, type 2 diabetes mellitus; CAD, coronary artery disease.

**Table 8:** Interactions of *FTO* rs17817449 polymorphism with obesity and other factors on the risk of T2DM and dyslipidemia.

Factors	Dyslipidemia			T2DM		
	TT genotype	TG genotype	GG genotype	TT genotype	TG genotype	GG genotype
Male, n (%)	287 (71.57)	107 (26.68)	7 (1.75)	146 (66.97)	63 (28.90)	9 (4.13)
Female, n (%)	132 (68.75)	55 (28.65)	5 (2.60)	72 (68.57)	32 (30.48)	1 (0.95)
*P*		0.41			0.46	
Smoking, n (%)	191 (74.32)	63 (24.51)	3 (1.17)	94 (65.28)	44 (30.56)	6 (4.16)
Non-smoking, n (%)	226 (68.07)	97 (29.22)	9 (2.71)	124 (70.06)	49 (27.68)	4 (2.26)
*P*		0.06			0.27	
Obesity, n (%)	56 (58.33)	37 (38.54)	3 (3.13)	25 (55.56)	18 (40.00)	2 (4.44)
Obesity-free, n (%)	331 (73.72)	109 (24.28)	9 (2.00)	163 (69.07)	67 (28.39)	6 (2.54)
*P*		< 0.01			0.08	
Dyslipidemia, n (%)	–	–	–	108 (66.67)	49 (30.25)	5 (3.08)
Dyslipidemia-free, n (%)	–	–	–	109 (69.87)	42 (26.92)	5 (3.21)
*P*		–			0.61	
Hypertension, n (%)	237 (70.54)	93 (27.68)	6 (1.78)	148 (68.52)	63 (29.17)	5 (2.12)
Hypertension-free, n (%)	180 (70.59)	69 (27.06)	6 (2.35)	70 (65.42)	32 (29.91)	5 (4.67)
*P*		0.90			0.39	
Hyperhomocysteinemia, n (%)	160 (72.07)	60 (27.03)	2 (0.90)	74 (69.16)	32 (29.91)	1 (0.93)
Hyperhomocysteinemia-free, n (%)	223 (68.83)	92 (28.40)	9 (2.77)	111 (66.87)	50 (30.12)	5 (3.01)
*P*		0.25			0.50	
Hyperuricemia, n (%)	149 (73.40)	51 (25.12)	3 (1.48)	66 (74.16)	20 (22.47)	3 (3.37)
Hyperuricemia-free, n (%)	265 (68.83)	111 (28.83)	9 (2.34)	150 (65.50)	72 (31.44)	7 (3.06)
*P*		0.19			0.22	
T2DM, n (%)	108 (66.67)	49 (30.25)	5 (3.08)	–	–	–
T2DM-free, n (%)	311 (72.16)	113 (26.22)	7 (1.62)	–	–	–
*P*		0.14			–	
CAD, n (%)	273 (70.91)	103 (26.75)	9 (2.34)	152 (69.09)	59 (26.82)	9 (4.09)
CAD-free, n (%)	146 (70.19)	59 (28.37)	3 (1.44)	66 (64.08)	36 (34.95)	1 (0.97)
*P*		0.97			0.77	

*FTO*, fat mass and obesity-associated gene; T2DM, type 2 diabetes mellitus; CAD, coronary artery disease.

## Discussion

The present study demonstrates that *FTO* rs9939609 and rs17817449 polymorphisms are significantly and independently associated with the susceptibility to dyslipidemia and T2DM in a Chinese Han population. The A allele of rs9939609 and G allele of rs17817449 polymorphisms confer a higher risk of dyslipidemia and T2DM. In addition, carriers of the A allele of the rs9939609 polymorphism or G allele of the rs17817449 polymorphism have a higher risk of developing dyslipidemia in obese patients than those in non-obese individuals. To our knowledge, this is the first time to demonstrate that the relations between the rs9939609 and rs17817449 polymorphisms and dyslipidemia are modulated by obesity.

In line with our findings, a series of studies have reported significant correlations of *FTO* rs9939609 and rs17817449 polymorphisms with the risk of T2DM and dyslipidemia (24, 28, 29, 33, 46–51). Younus et al. conducted a case-control study to investigate the impact of the rs9939609 and rs17817449 polymorphisms on the development of T2DM in a sample of obese Iraqis, and found that the minor alleles of both variants are associated with an increased risk of T2DM (33). In addition, the significant relation between the rs9939609 and rs17817449 polymorphisms and T2DM risk was reported in Vietnamese (28), Indians (29), Egyptians (47) and Czechs (48) and Pakistanis (51). Sierra-Ruelas and teammates explored the association of the rs9939609 variant with serum lipids among Mexican adults, and found that the A allele carriers had significantly higher levels of TC and LDL-C (24). Jalili et al. observed that the A allele carriers of the rs9939609 polymorphism had a lower average level of HDL-C than noncarriers in a cohort of Iranian women (49). In a population of Romanian Children, researchers demonstrated that the G allele carriers of the rs17817449 variant had higher levels of TC and TG (50).

As the rs9939609 and rs17817449 polymorphic sites are localized within *FTO*, a gene with the strongest association with obesity discovered so far, a large number studies have reported a significant association between the rs9939609 and rs17817449 polymorphisms and obesity among various populations including East and South Asians (52–58), Latin Americans (23, 59–61), Europeans (62), Africans (63) and Middle Easterners (64). The A allele of the rs9939609 polymorphism was also found to be associated with an increased risk of obesity in several cohorts of children respectively from Romania (50), China (65), Portugal (66) and Chile (67). These relations were further strengthened with a genome-wide association study (68) and a couple of meta-analyses (69, 70) which consistently demonstrated that the A allele of rs9939609 and G allele of rs17817449 polymorphisms confer a higher risk of obesity in children and adults. Somehow, no significant associations between the rs9939609 and rs17817449 polymorphisms and obesity were found in our study population. One of the reasons could be that the present study population was not made up of general or healthy individuals, but those people who came to hospital to see doctors with chest pain symptoms and suspected CAD. In fact, more than half of these subjects had CAD or hypertension, and close to half of them had dyslipidemia. In addition, quite a few subjects in this study had T2DM, obesity, hyperhomocysteinemia, hyperuricemia, dyslipidemia and other diseases. Similarly, Sylwia et al. also reported that the rs17817449 polymorphism is not related to obesity in a population of patients with CAD, diabetes mellitus, hypertension and dyslipidemia (27).

The results of this study indicated that *PPARD* rs2016520 and rs2267668 polymorphisms are not associated with any of the metabolic-related diseases including obesity, dyslipidemia, hyperhomocysteinemia, hyperuricemia, hypertension, T2DM and CAD. In agreement with our findings, several previous studies also demonstrated no associations between the genotypes of *PPARD* rs2016520 or rs2267668 polymorphism and the risk of obesity (41), dyslipidemia (41, 71, 72), T2DM (41, 73) or CAD (42). However, Jguirim-Souissi et al. (72) concluded that the rs2016520 polymorphism is an independent risk factor of CAD, although it was not found to be associated with plasma lipid levels either in CAD patients or in control subjects. Chehaibi and colleagues (71) obtained a similar finding that the C allele frequency of *PPARD* rs2016520 polymorphism was higher in stroke patients than in controls, but plasma lipid levels did not differ significantly between the genotypes. Two studies demonstrated the rs2016520 polymorphism is associated with both CAD and blood lipids (74, 75). Maculewicz et al. (39) found that the rs2267668 polymorphism is significantly associated with BMI. As mentioned above, there were numerous inconsistent reports in the scientific community regarding these two *PPARD* polymorphic sites and metabolic-related diseases. The main reason for these inconsistencies may be that PPARδ as well as its gene polymorphisms just have a weak impact on the metabolic-related diseases, so their relations are easily affected by environmental and other factors. Among the three members of PPAR family, PPARγ is most closely related to metabolic-related diseases, PPARα being the next, and both of them have been served as drug targets for clinical use (3).

The mechanisms underlying the correlations between *FTO* rs9939609 and rs17817449 polymorphisms and T2DM as well as dyslipidemia have not been fully elucidated. The first potential explanation could be that the rs9939609 and rs17817449 polymorphisms have led to aberrant expression of *FTO*, which in turn disturbs the methylation status of FTO-targeted mRNAs, resulting in abnormalities in glucose and lipid metabolism. Indeed, Villalobos-Comparán et al. observed that the A allele carriers of the rs9939609 variant expressed significantly more *FTO* mRNA than those with the TT genotype in biopsies of subcutaneous fat tissue of Mexican women (61, 76). Karra et al. (18) and Berulava et al. (77) had similar finding that the A allele carriers of the rs9939609 polymorphism had significantly more abundant *FTO* transcripts in skin biopsies and/or blood cells than those with the TT genotype. The target mRNAs of FTO, such as SREBP1c (10–12), ChREBP (12), PPARα (13), PPARγ (14) and PGC1α (15), are all well-known to be extensively involved in glucose and lipid metabolism. The second explanation may involve energy intake. Ghrelin is a stomach hormone that has been proved to stimulate appetite and increase energy intake (78). Karra et al. found that subjects with the AA genotype of the rs9939609 polymorphism had elevated *FTO* transcripts, decreased ghrelin mRNA m6A methylation, and increased ghrelin levels as compared to the TT homozygotes (18). Several other studies have also demonstrated that *FTO* polymorphisms are highly associated with appetite, satiety, and energy intake in children and adolescents (79–81). Therefore, the association between the rs9939609 polymorphism and T2DM or dyslipidemia may be mediated by excessive energy intake. The third explanation may be that the rs9939609 and rs17817449 polymorphisms work in concert with other genetic variants to affect the susceptibility of obesity, diabetes and hypertension (82–84). For instance, Sirtuin 1 (SIRT1), a histone deacetylase and an anti-aging gene, is involved in the regulation of PPAR with relevance to lipid metabolism in the adipose tissue and liver, and is associated with metabolic-related diseases, such as metabolic syndrome and cardiovascular disease (85, 86). *FTO* polymorphisms may interact with the genetic variants in *SIRT1* and other genes, and jointly regulate glucose and lipid metabolism.

There are several limitations to this study. First, the research population was not from general subjects, but a special group of people who had symptoms of chest pain came to the hospital for treatment and underwent coronary angiography examination. This may lead to sample-selection bias. Second, the research subjects are mainly made up of Chinese Han people, and hence the findings of this study may not apply to other ethnic origins. Third, the research adopted a cross-sectional design. It is necessary to carry out a follow-up investigation to determine the exact roles of the polymorphisms in *FTO* and *PPARD* in metabolic-related diseases.

## Conclusions

Minor alleles of *FTO* rs9939609 and rs17817449 polymorphisms confer a higher risk of T2DM and dyslipidemia, and the risk is further increased among obese individuals. Relations between *FTO* polymorphisms and T2DM as well as dyslipidemia are possibly mediated by abnormal glucolipid metabolism and increased energy intake.

## Data availability statement

The datasets presented in this article are not readily available because of ethical and privacy restrictions. Requests to access the datasets should be directed to the corresponding author/s.

## Ethics statement

The study protocol was reviewed and approved by the Ethics Committee of Chengdu University. The studies were conducted in accordance with the local legislation and institutional requirements. Written informed consent for participation in this study was provided by the participants or the participants’ legal guardians/next of kin.

## Author contributions

YS, YZ and LC conceived of the study, participated in the design, analyzed the data, and drafted the manuscript. LC, JZ and CH made the diagnosis of metabolic-related diseases. YZ, HL, LX and YW carried out DNA extraction, genotyping and collection of clinical data. SY revised the manuscript critically for important intellectual content. All authors reviewed and approved the final version of the manuscript for submission.
