# Supplementary material for: Minor alleles of FTO rs9939609 and rs17817449 polymorphisms confer a higher risk of type 2 diabetes mellitus and dyslipidemia, but not coronary artery disease in a Chinese Han population
Source: Front Endocrinol (Lausanne). 2023 Dec 15;14:1249070. doi: 10.3389/fendo.2023.1249070 (PMC10754952; doi:10.3389/fendo.2023.1249070)
Supplement: Supplementary file 1 [file Table_1.docx]

**Table S1.** PCR primers, amplification and digestion conditions.

| Polymorphisms | PCR primers | Product size | Amplification conditions | Restriction endonucleases | Genotypes |
| --- | --- | --- | --- | --- | --- |
| *FTO*  rs9939609 | F: 5’-CTAGGTTCCTTGC  GACTGCTGTGAACT-3’  R: 5’-TTCAAGTCACACT  CAGCCTCTCTACCA-3’ | 215 bp | 95℃ 7 min; 95℃ 30 s, 60℃ 30 s, 72℃ 30 s, 35 cycles; 72℃ 8 min | DdeI  C↓TNAG | TT: 215 bp  TA: 215 bp, 189 bp and 26 bp  AA: 189 bp and 26 bp |
| *FTO*  rs17817449 | F: 5’-GGAGTCTCCCCTT  AACTGGTC-3’  R: 5’-CACAGCAGGCATT  TACAAGCG-3’ | 430 bp | 95℃ 7 min; 95℃ 30 s, 60℃ 30 s, 72℃ 40 s, 35 cycles; 72℃ 10 min | AlwNI  CAGNNN↓C  TG | GG: 430 bp  GT: 430 bp, 357 bp and 73 bp  TT: 357 bp and 73 bp |
| *PPARD*  rs2016520 | F: 5’-ACCCACTACAAG  AGCCAGGT-3’  R: 5’-CAGTCATAGCTCT  TGCATCGT-3’ | 279 bp | 95℃ 7 min; 95℃ 30 s, 61℃ 30 s, 72℃ 35 s, 35 cycles; 72℃ 8 min | BslI  CCNNNNN↓N  NGG | TT: 279 bp  TC: 279 bp, 233 bp and 46 bp  CC: 233 bp and 46 bp |
| *PPARD*  rs2267668 | F: 5’-ACTAGAGGACGA  ATGGGTTTGG-3’  R: 5’-GATGTTAAGCAT  CTGTGGGACC-3’ | 109 bp | 95℃ 7 min; 95℃ 30 s, 61℃ 30 s, 72℃ 30 s, 35 cycles; 72℃ 5 min | HPY188I  TCN↓NGA | AA: 109 bp  AG: 109 bp, 78 bp and 31 bp  GG: 78 bp and 31 bp |

PCR, polymerase chain reaction; F, forward primer; R, reverse primer; *FTO*, Fat mass and obesity-associated gene; *PPARD*, peroxisome proliferator-activated receptor delta gene.
