# Supplementary material for: Minor alleles of FTO rs9939609 and rs17817449 polymorphisms confer a higher risk of type 2 diabetes mellitus and dyslipidemia, but not coronary artery disease in a Chinese Han population
Source: Front Endocrinol (Lausanne). 2023 Dec 15;14:1249070. doi: 10.3389/fendo.2023.1249070 (PMC10754952; doi:10.3389/fendo.2023.1249070)
Supplement: Supplementary file 2 [file Image_1.pdf]

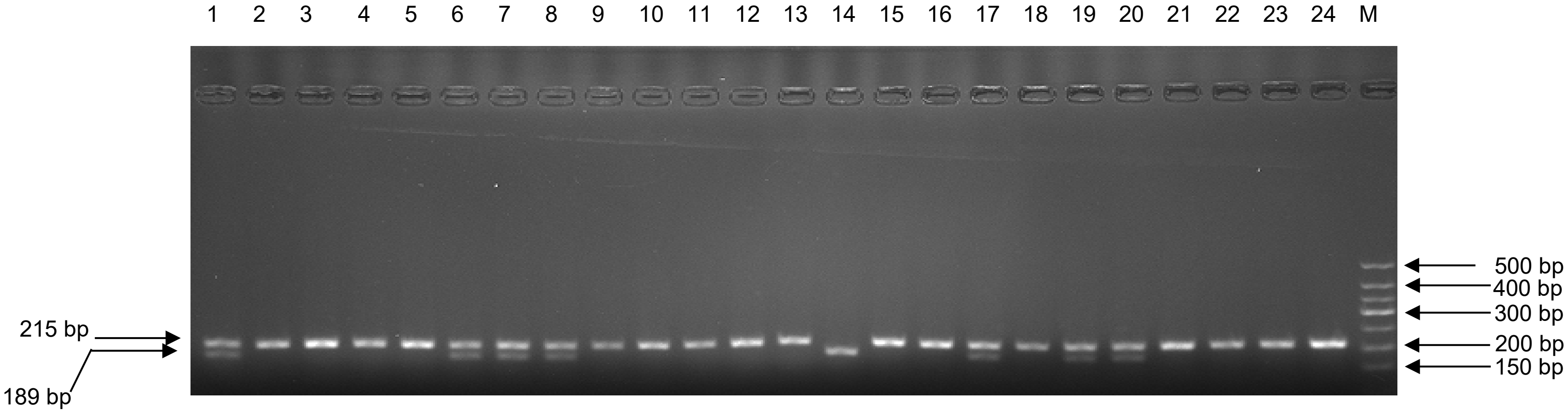

Figure S1. Gel electrophoresis map of the *FTO* rs9939609 polymorphism genotyped by PCR-RFLP method.

Lanes 2, 3, 4, 5, 9, 10, 11, 12, 13, 15, 16, 18, 21, 22, 23 and 24: TT genotype; Lanes 1, 6, 7, 8,17, 19 and 20: TA genotype; Lane 14: AA genotype.

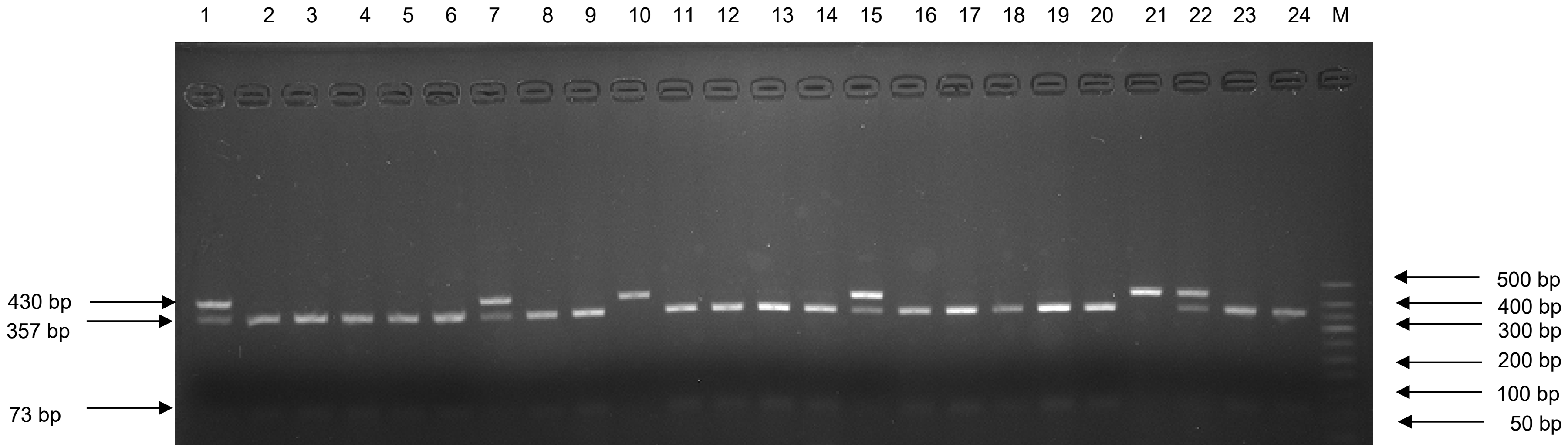

Figure S2. Gel electrophoresis map of the *FTO* rs17817449 polymorphism genotyped by PCR-RFLP method.

Lanes 2, 3, 4, 5, 6, 8, 9, 11, 12, 13, 14, 16, 17, 18, 19, 20, 23 and 24: TT genotype; Lanes 1, 7, 15 and 22: TG genotype; Lanes 10 and 21: GG genotype.

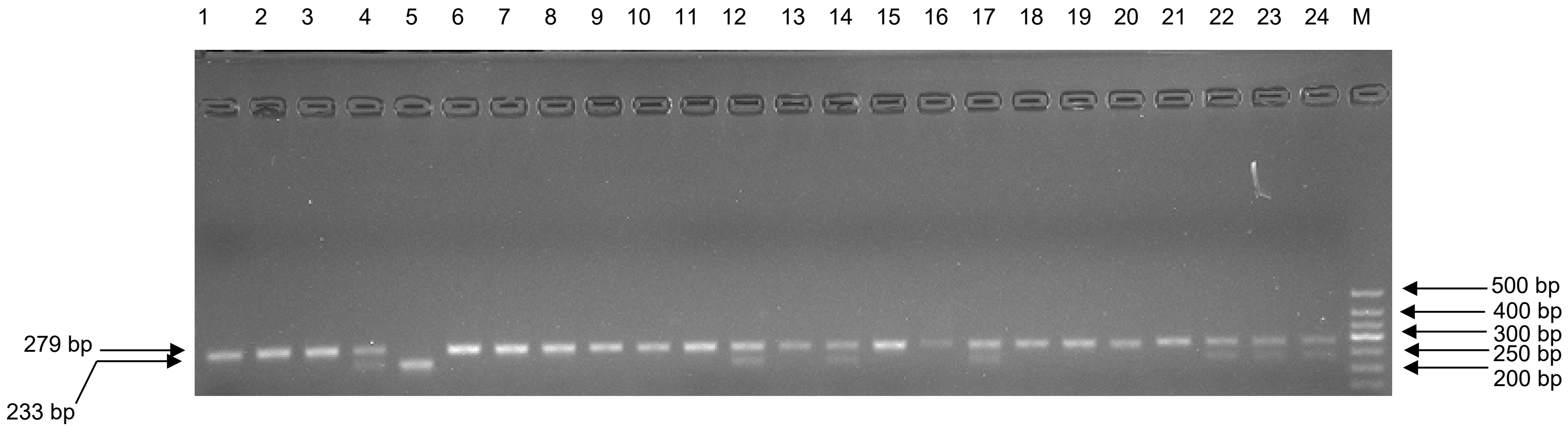

Figure S3. Gel electrophoresis map of the *PPARD* rs2016520 polymorphism genotyped by PCR-RFLP method.

Lanes 1, 2, 3, 6, 7, 8, 9, 10, 11, 13, 15, 16, 18, 19, 20 and 21: TT genotype; Lanes 4, 12, 14, 17, 22, 23 and 24: TC genotype; Lane 5: CC genotype.

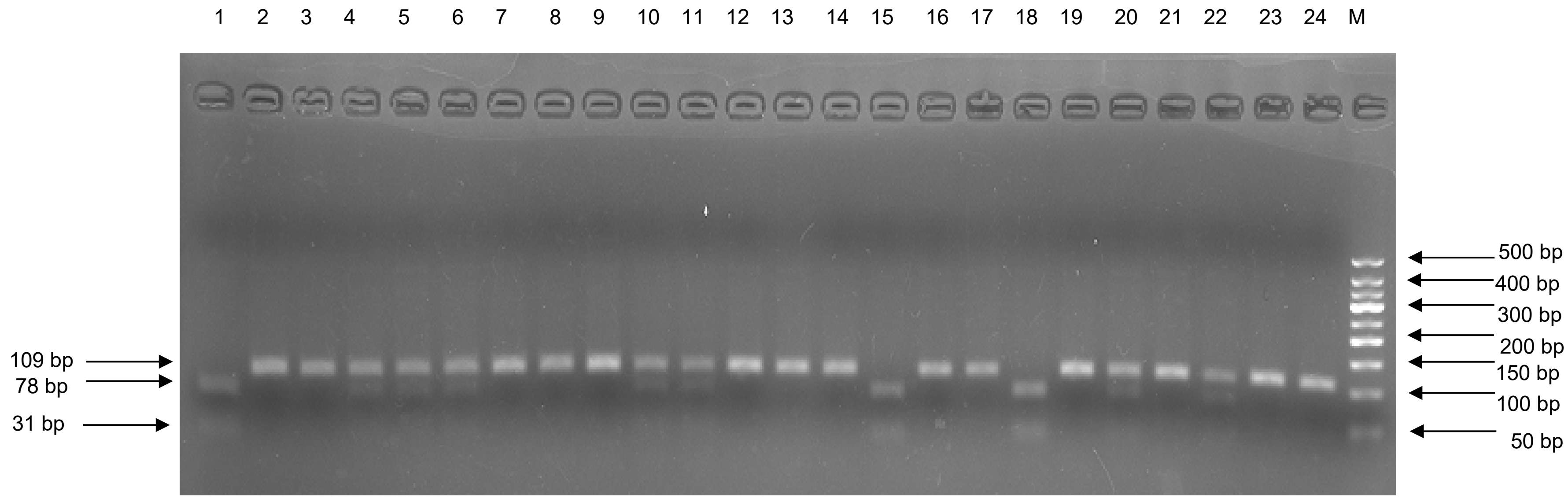

Figure S4. Gel electrophoresis map of the *PPARD* rs2267668 polymorphism genotyped by PCR-RFLP method.

Lanes 2, 3, 7, 8, 9, 12, 13, 14, 16, 17, 19, 21, 23 and 24: AA genotype; Lanes 4, 5, 6, 10, 11, 20 and 22: AG genotype; Lanes 1, 15 and 18: GG genotype.
